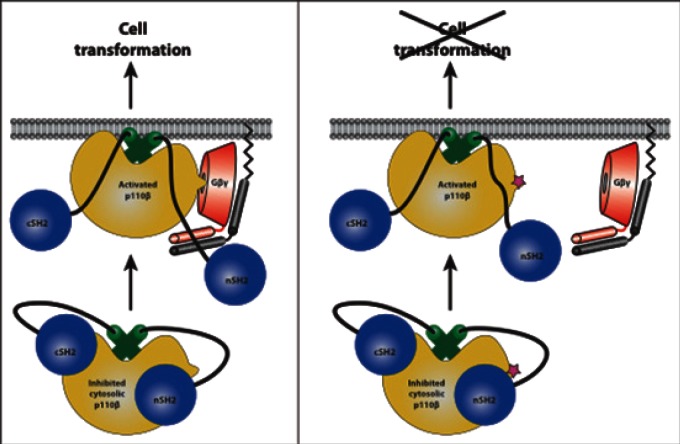# PI3Kβ downstream of GPCRs – crucial partners in oncogenesis

**DOI:** 10.18632/oncotarget.787

**Published:** 2012-12-18

**Authors:** Hashem A. Dbouk, Oscar Vadas, Roger L. Williams, Jonathan M. Backer

**Affiliations:** Dept. of Molecular Pharmacology, Albert Einstein College of Medicine, Bronx, NY; MRC Laboratory of Molecular Biology, Cambridge, UK; MRC Laboratory of Molecular Biology, Cambridge, UK; Dept. of Molecular Pharmacology, Albert Einstein College of Medicine, Bronx, NY

Of the class IA PI3Ks, p110β is the only member to simultaneously signal downstream of both Receptor Tyrosine Kinases (RTKs) and G Protein-Coupled Receptors (GPCRs). While the mechanism of RTK-mediated activation of p85/p110 heterodimers has been characterized [1, 2], defining the mechanism of Gβγ-mediated activation of p110β downstream of GPCRs has been challenging due to the transient nature of the interaction. The binding of p110β to Gβγ is weak, but is reinforced by interactions with lipid membranes, where Gβγ resides. A combination of sequence analysis and hydrogen/deuterium exchange (HDX) coupled to mass spectrometry (MS) experiments has enabled us to map the regions of interaction on both p110β and Gβγ [3]. Furthermore, HDX-MS data analysis provided a unique insight into the mechanism of p110β activation by Gβγ, showing that p110β kinase domain makes stronger interaction with membranes in the presence of Gβγ. The regulatory p85 subunit inhibits basal activity of p110β, largely by the inhibitory contacts that the nSH2 and the cSH2 domains of p85 make with the p110β subunit [1, 2]. The nSH2 contacts the helical domain of p110β close to the residues essential for Gβγ binding. Whether the nSH2 has to disengage from p110β to allow Gβγ binding is still uncertain. In vitro, p110β/p85 truncation constructs lacking either the nSH2 or both SH2 domains are still activated by Gγ. In contrast, activation of p110β/p85 constructs by tyrosyl phosphorylated phosphopeptides require the presence of at least one p85 SH2 domain. Thus, Gβγ and phosphopeptides activate the enzyme via distinct mechanisms, resulting in synergistic activation when both activators are present. Whether there is an allosteric component of activation in addition to membrane recruitment mediated by Gβγ remains an open question. Based on the current results and on the structure of a membrane interacting heterotrimeric G-protein, we can begin to model how the activated p110β would interact with membranes. In this model, Gβγ makes no direct contact with the kinase domain of p110β, suggesting a mechanism where Gβγ stimulates p110β activity by increasing its membrane residence time. A similar mechanism has been proposed for the other Gβγ-sensitive PI3K, p110γ [4]. The crystal structure of p110β in a complex with Gβγ will answer many remaining questions.

Wild-type p110β is transforming when over-expressed in fibroblasts [5], and it is the major p110 isoform required for driving PTEN^−/−^ tumors [6]. However, it has not been previously possible to determine whether GPCR activation of p110β/p85 was required in these cases. Using a mutant p110β or a cell-permeable peptide, both of which block GPCR- mediated but not RTK-mediated activation of p110β/p85, we showed that GPCR inputs to p110β/p85 are required for transformation, proliferation, chemotaxis, and invasion driven by either p110β over-expression or stimulation with GPCR ligands [3]. Furthermore, a requirement for GPCR activation of p110β/p85 was seen in the growth of PTEN^−/−^ cell lines, but not PTEN^+/+^ cells. Surprisingly, the peptide inhibitor of p110β/Gβγ binding blocked the growth of PTEN^−/−^ cells, whereas the p110β-specific kinase inhibitor, TGX-221, did not. This suggests a role for a Gβγ-mediated scaffolding function of p110β in proliferation. This is consistent with previous studies showing that some functions of p110β are kinase independent [7, 8]. Our work suggests that in some tumors, inhibitors specifically targeting the Gβγ-p110β interaction might be more potent than inhibitors targeting p110β catalytic activity. Our study also suggests that the identification of the GPCRs that drive PTEN^−/−^ tumors could provide an important alternative therapeutic approach for the treatment of these tumors. Understanding the regulation of p110β catalytic activity, as well as defining its scaffolding functions, will be important in developing drugs that target its functions in human disease.

**Figure:**